# Rapid Review on COVID-19, Work-Related Aspects, and Age Differences

**DOI:** 10.3390/ijerph18105166

**Published:** 2021-05-13

**Authors:** Lara Bellotti, Sara Zaniboni, Cristian Balducci, Gudela Grote

**Affiliations:** 1 Department of Psychology, University of Bologna, 47521 Cesena, Italy; lara.bellotti1995@gmail.com; 2 Department of Management, Technology, and Economics, ETH Zürich, 8092 Zürich, Switzerland; ggrote@ethz.ch; 3 Department of Psychology, University of Bologna, 40127 Bologna, Italy; cristian.balducci3@unibo.it

**Keywords:** coronavirus, work, age, occupational risk, labor market, retirement, remote work, individual and organizational strategies

## Abstract

The COVID-19 pandemic significantly impacted the labor market and multiple aspects of work and workers’ life. The present rapid review analyzes this impact considering the effects that COVID-19 pandemic had on employment and work-related aspects across different age groups. A comprehensive literature search was performed on scientific contributions published between 2019 and March 2021, resulting in 36 papers pertinent to the scope of this review. Findings were grouped according to different topics, all linked to age: occupational risk, implications on the labor market (i.e., job loss and reemployment, job insecurity, turnover intentions and retirement, and healthcare workers’ return-to-work phase), remote work, and key individual and organizational resources and strategies. Overall, the review revealed variability across age groups in the impact this pandemic had on employment and several work-related aspects (i.e., occupational risk, remote work). Findings supported an age-differential effect of normative history-graded events such as the current pandemic, highlighting different responses and consequences depending on workers’ age.

## 1. Introduction

COVID-19 was assessed to be a pandemic by the World Health Organization (WHO) on 11 March 2020 [1]. Since then, this ongoing crisis has not only dramatically affected public health and the worldwide economy, but also significantly impacted the labor market and multiple aspects of work and workers. On the one hand, the pandemic disrupted the labor market by increasing unemployment levels worldwide [2]. On the other hand, it also contributed to the adoption of innovative ways of work, such as remote work [3].

The impact of this pandemic on people’s work sphere can be analyzed while looking for differences as well as similarities between individuals of different age groups [4]. In fact, different types of life events can trigger age-differential responses: normative age-graded influences (i.e., common biological changes or contextual events such as marriage), non-normative influences (i.e., unpredictable personal events such as severe illness and contextual events such as divorce), and normative history-graded influences (i.e., events tied to a specific historical period, such as the Great Depression or pandemics) [5,6]. The way these events are experienced may vary with age. In the work context, it is important for individuals, organizations, and stakeholders (e.g., policy makers) to understand age-related issues in response to these major events.

Reviews on the psychological outcomes of the pandemic and its impact on the healthcare sector have already been published (e.g., [7,8,9,10]). However, so far there are no reviews focusing on the impact of this crisis on employment and work-related aspects using a lifespan perspective (i.e., comparing younger and older workers’ responses to different areas of employment and work). The present contribution addresses this gap, reporting a comprehensive analysis of the consequences of the pandemic on multiple aspects of work and workers.

Firstly, since COVID-19 is a disease spreading through airborne transmission, we highlighted the definition of occupational-risk in the context of the pandemic. Patterns of risk are analyzed in relation with workers’ age [11,12,13]. Secondly, we explored the impact of the pandemic on the labor market and work. To reach this end, we considered the share of job losses across the population and the changes in remote work. These phenomena are thought to elicit different reactions across workers of different age. For example, the high rate of unemployment is likely to trigger perceptions of job insecurity, especially across those workers with the greater probability to lose their job. In situations of perceived insecurity, workers are more likely to exit their jobs to deal with the stress elicited by high uncertainty (i.e., rather than not knowing what will happen, individuals take initiative and create predictability) [14]. Unemployment is among the key antecedents of job insecurity [15], which in turn has been positively linked with turnover intentions [16,17]. We investigated each concept (i.e., unemployment, job insecurity, and turnover intentions) highlighting age-related differences. Moreover, we explored the interaction of the pandemic with individual and contextual factors promoting different retirement decisions across older workers (i.e., anticipating retirement, postponing retirement, or stepping back from retirement). Thirdly, we emphasized the consequences of the pandemic on remote work and its advantages and disadvantages, especially in relation to occupation and age-related challenges. We also highlighted the role of age in the context of virtual collaboration and its social-related aspects. Lastly, we presented contributions underlying the individual resources useful to deal with the COVID-19 consequences and the strategies that organizations can adopt to promote these resources and offer supportive contextual factors. We reported age differences in responses and usefulness of different strategies. Understanding such strategies is important to inform policymakers and corporations on possible actions to limit the disruptive effect of the pandemic and keep individuals motivated to work.

Therefore, the scope of this review is to analyze the different scientific contributions on the effects of the COVID-19 pandemic in respect to different facets of work (i.e., occupational risk, the labor market, job insecurity, turnover intentions, retirement, remote work, and personal and contextual resources/strategies), while highlighting age-related differences, when present.

## 2. Materials and Methods

The study was based on a rapid review approach following the Cochrane Rapid Review recommendations [18]. Rapid reviews are a type of review that is simultaneously satisfying the time-sensitive needs of policy makers and the scientific need of methodological quality [18,19].

The present rapid review was performed according to these steps: (i) framing of the explorative research question using the PICO framework (i.e., Problem, Intervention, Comparison, Outcome), (ii) development of the search strategy, (iii) specification of inclusion criteria, (iv) abstract screening and study selection, (v) data extraction, and (vi) synthesis of findings.

The identified research question is the following: What effects did the COVID-19 pandemic have on employment and work across different age groups?

A comprehensive literature search was performed using the database *Web of Science*, *Scopus*, and *PsycINFO*. The following Boolean expression was entered: (covid* OR coronavirus OR pandemic) AND (age OR aging) AND (work* OR workplace OR retir*).

The articles had to be published between 2019 (i.e., beginning of the pandemic) and April 2021 (i.e., date of last search 11 April 2021) and in English. No limitations were made on the type of paper (i.e., empirical contributions, commentaries, reports, working papers, perspectives).

A total of *n* = 4768 articles were found. After removing the duplicates, the abstracts and full-texts were screened. Papers targeting different aspects of work/work context/workforce/labor market and workers’ age in the context of the pandemic were selected. Papers mentioning the COVID-19 pandemic in the recommendation section only were excluded, and thus not explored/analyzed in the contribution. All studies focusing on the consequences of the pandemic solely on health (e.g., physical health, mental health) were excluded as well. In fact, these topics are already subject of other reviews [7,8,9,10].

**Figure 1:**
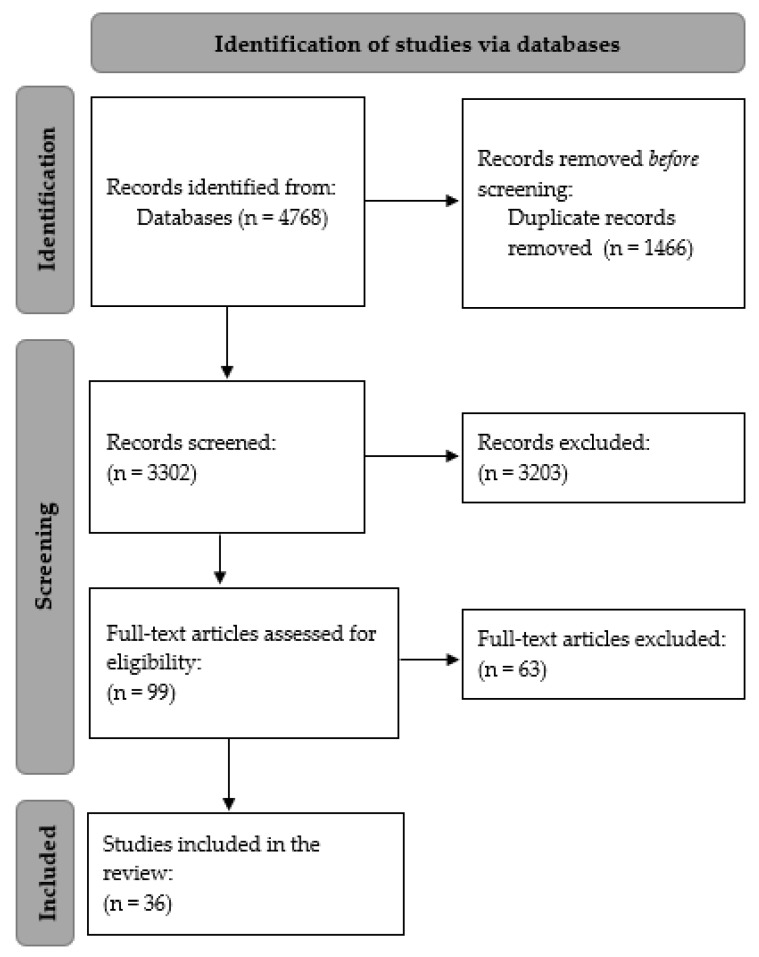
Flowchart of the study selection process for the Rapid Review.

Finally, *n* = 36 articles were identified from the literature, of which *n* = 17 were empirical and *n* = 19 were commentaries or perspectives (Figure 1).

Findings were grouped in the results’ section according to different topics, all linked to age: occupational risk, implications on the labor market (i.e., job loss and reemployment, job insecurity, turnover intentions and retirement, and healthcare workers’ return-to-work phase), remote work, and key individual and organizational resources and strategies. Several papers crossed different subsections. Table 1 presents a summary of the main aspects (i.e., author(s), contribution type such as empirical or commentary/perspectives, data type and source when empirical contributions, country, and key results) and subtopics for each paper selected for the rapid review. Papers located in multiple subsections are highlighted in gray.

## 3. Results

### 3.1. Occupational Risk and Age

Since its beginning, individuals from different occupations and age groups have been differently hit by the pandemic. Hence, specific categories of workers were risking more than others mainly because of how the contextual (e.g., occupation-specific risks related to the conditions in which the job is performed) and individual (e.g., age) factors interact, increasing the probability to get infected.

When we refer to occupational risk in the context of COVID-19, we used the definition provided by O*Net [20]. Accordingly, there are two components: (i) the level of physical proximity to other individuals and (ii) the frequency of exposure to possible diseases or infections. Several researchers found that the individuals with the highest risk for infection are those employed in low-income and low-skill occupations (e.g., construction), healthcare workers, and service workers (e.g., transportation, retail trade, hospitality) [20,21,22]. For example, more than 95% of Canadian low-income workers were found to be employed in jobs belonging to the top half of the physical proximity score distribution of COVID-19 [20]. Hoehn-Velasco and colleagues [21] and Kim and colleagues [22] also acknowledged a higher risk of infection for Mexican hospitality workers such as hotel employees, and South Korean healthcare employees such as nurses respectively.

At the same time, the COVID-19 pandemic had a heterogeneous impact on different age groups. Indeed, worldwide findings suggested that the mortality rate grew exponentially for those aged more than 50 years old [23,24,25,26,27]. For instance, a study on the Chinese population indicated that while the mortality rate was less than 0.5% for people aged less than 50, it rose to 1.3% for those aged between 50 and 59 and escalated up to 3.6% for those 60 to 69 [24]. Similar results were found in the U.S. [26]—where mortality rate jumped two to four times when the 60-years age group was compared to the 70-years one. Estimates from U.K. (April 2020) data showed that, for 1 million infections, 70% of all deaths would be among people aged 70+, and the 64% of the remaining deaths would be in the 60–69-years age group [25].

In examining the consequences of the pandemic, some scholars accounted for the effect of the COVID-19 pandemic on both dimensions of age and occupational risk taken together, suggesting that occupation-specific risks and age-related risks will therefore increase the overall individual risk when interacting [24,27,28].

In their commentary, Kanfer and colleagues [27] suggested that the overall health risk for older workers employed in low-income occupations is dramatically high when compared to situations of employment in high-income jobs. This happens because oftentimes, low-wage jobs are also considered “essential” such as healthcare workers with less opportunities to work from home to lower their risk of infection.

To assess the combinatorial risk of age*occupation, Larochelle [24] proposed a theoretical framework accounting for (i) the occupational-specific risk of contracting the disease, and (ii) the likeability of death depending on age (in addition to the presence of chronic conditions). Both risks can be categorized as low, medium, or high. This risk’s assessment is served by a counselling guideline: for instance, individuals scoring high in both domains should be made aware on the high risks associated to their work activity and may consider work from home. Individuals scoring high on one risk and medium on the other should be advised on risks and opportunities to mitigate exposure. Lastly, individuals located in the remaining combinations (e.g., low risk of death and high occupational-specific risk, or medium or low in both) should be instructed on how to follow the WHO guidelines (i.e., wear a mask outdoors, hand hygiene, use of personal protective equipment).

In light of these observations, it seems important to protect aged, vulnerable workers and understand the worker-occupation relationship. In this respect, one study contributed to shed light on whether high-risk occupations are mostly held by younger or older workers. St-Denis [20] performed quantitative analyses on the relationship between age and occupational risk among Canadian workers and found that older workers (55+ years old) were not employed in occupations with higher scores of physical proximity compared to younger ones. In fact, younger people (15–24 years old) scored the highest. However, results from the same study also highlighted the opposite trend among healthcare workers, with workers aged 55+ displaying the highest risk for frequency of exposure than their younger colleagues. Hence, health workers’ infections rates were higher than in any other occupational group [20].

Specifically, jobs performed in the healthcare sector oftentimes require multiple interactions with several people, a notable duration of exposure, and the adoption of practices that might put them at risk (e.g., reduced social distancing) [20,26]. Data on healthcare workers mortality rates when accounting for age are therefore appalling: in Italy, 74 doctors in their 60s died of COVID-19 by April 2020 [23]. In the U.S., although among the healthcare workers only 6% of the infected were 65 years or more, they still represented 37% of deaths [26].

To summarize, the few existent studies called attention to seemingly correlated patterns, such as intertwined effects between COVID-19, occupation-specific risks, and the role of age. Several researchers discussed the importance of these intertwined effects to ensure fair working conditions for the most vulnerable workers. In fact, global evidence showed a greater COVID-19-related risk (i.e., higher mortality rate) for people aged more than 50-years old. Such aforementioned age-related risk is amplified when older workers are also employed in occupations with higher physical proximity to other people and frequency of exposure to potential pathogens (e.g., construction, healthcare).

### 3.2. Labor Market Implications and Age

#### 3.2.1. Job Loss, Reemployment, and Age

According to a recent ILO report [2] on latest labor market developments, the pandemic has led to an unprecedented global-level job loss, estimated at 114 million jobs. Data from different countries support this report: in Mexico around 1.1 million of formal jobs were lost in the first five months of the pandemic [21], in the United States around 20% of the active population applied for unemployment benefits in spring 2020 [27], and in Portugal the unemployment rate from the 4th quarter of 2020 to the 4th quarter of 2019 has raised by 0.4 percentage points [29].

Overall, employment loss was uneven, differentiating for income, job skills, and age. Research revealed low-income low-skill jobs, and temporary workers to be the most impacted [21,29,30,31]. For example, in Mexico the formal job’s market contracted by 5.4% in the first nine months, with the construction sector as the most affected [21]. A study by Kikuchi, Kitao, and Mikoshiba [30] found that in May 2020, Japanese service workers (e.g., transportation, retail trade, hospitality) saw the greater decrease, with more than a 5% drop in the employment rate. It is important to note that in Japan the general unemployment rate held stable at 2.9% in the same period.

Moreover, variability was found among workers of different age as well. Specifically, research showed that unemployment increased across all age groups but with different patterns. Younger workers displayed the higher share of job loss from the beginning of the pandemic [30,31,32], but appeared to rebound quickly with more stable job loss rate in the last months of the pandemic [21,29,33]. However, older worker job loss rates in general were lower compared to younger, but did not stabilize and, once they lost their jobs, they are thought to face more difficulties in finding a new occupation [21,27,29,31,33,34,35].

Going more in detail on studies on the job loss share, it has been shown that younger workers lost more jobs than older workers [30]. A study on Bangladeshi youth (age range 15–29) showed that about 5% of people lost their current job, almost 10% lost one of their current jobs, and approximately 9% saw their job offers postponed [32]. Moen and colleagues [31] analyzed data collected in the United States from the Current Population Survey (i.e., a monthly U.S. labor force survey, provided by the Integrated Public Use Microdata Series and including demographics and employment data, among others [36]). On the one hand, people in their 20s experienced the highest increase in their unemployment scores, equal to 9.4%. On the other hand, people in their 60s reported a 4.4% increase in unemployment.

Despite their higher job loss share, younger workers seem to be able to recover quicker (e.g., after the first months of vertiginous drop, the unemployment rate stabilized) than older workers. Data from the Mexico’s Social Security Institute [21] showed that younger workers’ job losses stabilized within the first six months of the pandemic. Workers over 60 instead, kept losing their jobs and by November 2020, they lost more jobs than any other age group [21]. Further data from the U.S. Bureau of Statistics showed that workers aged 65+ saw a 2.9% increase in their unemployment rate from April 2019 to April 2020 [34].

This is important because, as several scholars suggested, older workers face more difficulties in finding a new occupation in case of COVID-19-related unemployment [27,29,31,33,34,35]. Monahan and colleagues [34] reported results showing a general lower likeability of people over 50 to be rehired compared to younger individuals, and a higher probability to be reemployed into lower paid positions. Similar findings were reported after the economic recession in 2008: workers aged 62+ were the least likely age group to become reemployed once they lost their jobs [33]. A possible explanation suggested is that older workers are perceived as more vulnerable and frailer (e.g., higher mortality rate), and their opportunities for employment after the pandemic are negatively affected [31,35].

To summarize, the COVID-19 pandemic directly impacted the labor market, determining heterogeneous job losses among workers from different occupations and age groups. Different pathways for younger (e.g., higher share of lost jobs but quicker recovery) and older workers (e.g., lower job loss’ share higher barriers for reemployment) were observed.

#### 3.2.2. Job Insecurity and Age

One of the most serious consequences of the pandemic has been the growth of job insecurity, fostered by the increased levels of unemployment [29,37]. In fact, job insecurity refers to people’s own perceptions of potentially losing their jobs, it implies a degree of uncertainty about one’s future [15], and is triggered by the presence of threats to the stability of the labor market [38].

Since job unemployment is among job insecurity’s antecedents [15,29,37] and younger individuals displayed the higher share of job loss [31,32], we can expect that younger workers show higher levels of job insecurity in the COVID-19 pandemic. Two studies investigated this research area. In a study among 624 Serbian workers, Bajrami and colleagues [37] found a significant negative correlation between age and job insecurity (*r* = −0.29, *p* < 0.05), suggesting higher levels of the latter for younger workers. The same trend was found by Ranta and colleagues [39] on 222 young Finns (age range 18–29) participating in a national study, that appeared to be significantly more worried (∆M = 0.56, *p* < 0.001) about the consequences of the pandemic on their career than the rest of the Finnish population (30–65).

Given the limited number of studies delving into this topic, the evidence is not conclusive. However, a possible explanation for age differences in job insecurity levels is offered by Grote and Pfrombeck’s [40] commentary on uncertainty management in aging. Accordingly, older workers might better capitalize the opportunities arising from uncertain circumstances by engaging in either opening or closing behaviors. Opening behaviors have a proactive component that promotes uncertainty as a source of opportunity (e.g., changing one’s job), while closing behaviors are aimed to reduce uncertainty to act in a known environment (e.g., choosing to perform a familiar task) [40]. The authors suggested that by regulating the amount of uncertainty, perceptions of future opportunities are either increased or reduced. This greater capacity to manage uncertainty might be due to older individual’s better emotional regulation [40,41]. Therefore, since uncertainty is a main component of job insecurity [15], it is conceivable that juniors will engage less in self-regulatory uncertainty management processes, tightening their vision of the future along with their expectations for development opportunities, and ultimately increasing job insecurity [40].

#### 3.2.3. Turnover Intention, Retirement, and Age

The effect that labor market conditions had on workers’ perceptions, such as job insecurity, also expanded to workers’ intentions to continue working or to exit the labor market. Differences were found when age was considered. In fact, researchers suggested that job insecurity, triggered by this pandemic and exacerbated by the unpredictability concerning both the extent and strength of the crisis [37], can affect people’s intentions to leave their jobs (i.e., turnover intentions) [29,37]. In this scenario, younger workers displayed higher levels of turnover intentions when compared to their older counterpart [22,37,42,43].

Several studies supported this phenomenon. Findings from a cross-sectional study on Serbian workers [37] indicated that increased levels of job insecurity during COVID-19 induced higher turnover intentions. Moreover, the authors found that older participants (40+) had lower intentions to leave their organization and a stronger job motivation when compared to younger employees [37]. Similar findings were found across different countries (i.e., South Korea, Bolivia, Peru): workers’ age was negatively related with turnover intentions, therefore older employees were more likely to stay compared to younger ones [22,42,43]. 

Nonetheless, scholars have highlighted the role of individual related aspects (i.e., work ability, job importance, perception of threat) [27,28,34,44] and contextual related aspects (i.e., perceptions of discrimination, fewer hiring and reemployment opportunities, financial situation) [28,31,32,45] that might influence older workers’ intentions to exit the workforce.

With regards to the individual factors, Truxillo and colleagues [44] suggested that the likelihood of older workers to exit the workforce and retire earlier is influenced by the degree of negative impact that COVID-19 has on work ability (i.e., a person’s ability to continue working, considering the physical, mental, social, environmental, and organizational demands of their job). Kanfer and colleagues [27] hypothesized that decisions surrounding retirement depends on whether workers derive their meaning of life from work or from another life sphere, such as their family. Other authors argued that older workers’ resolutions to anticipate retirement are prompted by the degree to which returning to work is seen as potentially life-threatening (i.e., how likely they feel they will get infected and feel severely ill) [28,34].

Going more in detail on the contextual factors, Moen and colleagues [31] questioned whether it is possible that perceptions of discrimination and fewer opportunities for hiring push older workers to exit the workforce permanently. As highlighted by van Dalen and Henkens [45], in most developed countries workers already struggle to save an adequate amount of money for their retirement. It is possible that, to survive the COVID-19 economic consequences, workers might be forced to draw from their retirement saving, worsening their financial security [33]. For instance, Biggs [46] analyzed the way the Social Security Administration’s (SSA) measure of average wages interacts with the Social Security benefit formula, and found that the sudden decline in the average United States earnings in 2020 could lead to huge (−9%) reductions in Social Security retirement benefits, especially for those workers born in 1960 and after. Therefore, unplanned retirement might actually be an uncomfortable option and older workers may prefer postponing retirement [28,33,34].

Overall, data collected on retirement are contradictory, even within the same geographical area and timeframe: in Monahan and colleagues’ [34] commentary, findings from a U.S. large-scale households’ survey conducted in April 2020 reported a 7% increase in early retirement as a result of the pandemic. This is line with evidence collected after the economic recession in 2008, where half of the people aged 62 and older in the U.S. decided to drop out of the labor force and chose to retire after nine months of unemployment [47]. However, U.S. data from the monthly Current Population Survey (Integrated Public Use Microdata Series, [36]) collected until April 2020 showed that so far, there has been little increase in retirement as a consequence of the COVID-19 pandemic [31]. The only exception occurred among Asian men in their 60s and 70s without a college degree, that experienced a 6.6 and 8.9 percentage points increase in filed early retirement requests [31]. According to the authors, this might be due to their higher likelihood to exit fulltime jobs and transition to unemployment during the pandemic (13.2% increase for Asian men in their 60s, and 12.4% for those in their 70s). Therefore, it is conceivable the role that additional individual and contextual factors might have in orienting workers’ employment decisions. As several scholars hypothesized, it is conceivable that these effects might be better understood on the long term [27,29,31,33,34].

In sum, job insecurity has been linked to employees’ decisions whether to continue working. Evidence suggested greater turnover intentions among younger workers. However, further individual (i.e., work ability, job importance, perception of threat) and contextual (i.e., perceptions of discrimination, fewer hiring and reemployment opportunities, financial situation) factors impact older workers’ intentions to either anticipate or postpone retirement, and therefore leave or stay in their jobs.

#### 3.2.4. Retired Healthcare Workers’ Return to Work

The COVID-19 pandemic also disclosed new opportunities in the labor market, such as job openings targeting older and retired workers in specific occupations. More in detail, the growth of the pandemic and the subsequent rise in the number of patients in hospitals has pushed governments to call for retired healthcare professionals to voluntarily step out of retirement and return to work [26,48,49]. Many have answered the call as suggested by data from Canada (e.g., over 3000 nurses in the first two weeks of March 2020 [50]), the U.S. (e.g., 1000 NYC doctors only during the first day of call in March 2020 [51]), the U.K. (e.g., nearly 34,000 doctors by February 2021 [52]), and Italy (e.g., over 7000 doctors in March 2020 [53]) [49].

Considering the high occupational risk of infection of health occupations, these workers are facing additional risk due to their age [26,48,49]. For example, data from the U.S. population showed that although only 6% of infected healthcare personnel were 65 years or older, they still represented 37% of deaths [26].

Therefore, it is preferable for older and retired health workers to manage non-COVID-19 patients or avoid “patient-facing” roles, by providing telehealth consultations [26,48]. In fact, Peisah and colleagues [48] stressed the importance to promote a transition to online work also for health occupations (e.g., late-career and retired doctors or nurses): roles requiring telephone triage, assessment of symptomatic patients, and tele-health provision can be adapted to working from home and therefore suitable for late-career and retired workers. Hence, the pandemic expanded the opportunities for the implementation of remote work in high-occupational-risk sectors such as the healthcare.

### 3.3. Remote Work and Age

Since the pandemic outbreak, lockdowns were put in place and non-essential businesses closed to control the transmission of the virus. As a result, the proportion of workers able to carry out their tasks from home increased globally [54]. In Lithuania, workers applying telework swelled to 40% during the lockdown, compared to 13% in 2017 [55]. In the U.S., the number of people working from home reached approximately 49% by April 2020 [27]. Remote work has several advantages: for instance, it increases organizations’ agility and flexibility, attracts talents from all over the globe, and saves costs for real estate as offices are less needed [55]. During a pandemic, these are essential capabilities to survive the economic crisis, continue to fuel the labor market, and at the same time, allow workers to stay safe [45,55]. Moreover, working from home reduces commuting, which is in favor of the environment [29].

Nevertheless, remote work also comes with some disadvantages. For example, it cannot be applied equally across all occupations: this, in turn, can aggravate existing inequalities [29,56]. Furthermore, heterogeneous results on work from home opportunities, such as differences across occupations, and on how the experience of remote work is perceived has been found across workers of different ages [34,56].

With regards to the variability across occupations, a recent study on the Portuguese context highlighted that some low-income workers such as construction workers, faced relevant struggles (e.g., limited possibilities to switch certain job tasks online, limited or outdated equipment) transitioning their work online [29]. Results from Canada’s Employment Income Statistics [56] showed that while approximately 41% of jobs in Canada have the potential to be done from home, low-income jobs such as seasonal, contractual jobs (e.g., agriculture, construction) displayed a lower likeability to be performed from home.

Opportunities for remote work depend on one’s own occupation and may have a different impact across age groups [34,56]. Gallacher and Hossain [56] found that among the Canadian population, younger individuals were those more likely to be employed in those jobs where remote work is less possible, such as manual jobs (e.g., construction), and in turn, younger workers were those with the lower levels of remote work opportunities. However, Monahan and colleagues [34] showed the opposite result. Analyzing data from the U.S. Bureau of Statistics, they found that older workers tended to be employed in manual jobs for which remote work is hard to apply and estimated that in April 2020 in the U.S. only 30% of older adults (aged 65+) were working from home, compared to the 40% of younger ones (aged 25–34).

Moreover, age also influences how the experience of remote work is perceived. Raišienė and colleagues [55] asked 436 Lithuanian employees to evaluate remote work efficiency by rating different factors and found that while older workers tended to emphasize remote work’s disadvantages, younger employees mostly highlighted the advantages (e.g., time saved on commuting) and needed competences (e.g., good time management skills). Examples of the salient issues raised by older workers are the lack of direct feedback and interactions with manager and colleagues, higher work-life conflict issues, greater emotional burden [55]. Further findings from Portillo and colleagues [57] among 4589 Basque teachers showed that older teachers, when asked about remote teaching, generally felt less technologically competent than younger ones.

Lastly, some authors argued that a prolonged remote work situation might have adverse consequences on workers’ belonginess needs, quality of coworkers relationship, and social isolation perceptions, which are particularly relevant factors especially for older workers [34,45].

To sum up, the pandemic has been the prelude for a notable growth of remote work across different countries. Despite its advantages (e.g., extending talents’ attraction, real estate saved costs), there are also several aspects to consider in relation to occupation-specific and age-related challenges (e.g., heterogeneity in how remote work is applied across occupations, exacerbation of existing inequalities).

#### Social Aspects in Virtual Workplaces

The transition to remote work has also created new virtual spaces for collaboration, with unique opportunities (i.e., age diversity can stimulate innovation, possibility to work despite the time zone, increased productivity) [55,58] and challenges (e.g., work-life conflict, satisfaction of belonginess motives, the exacerbation of existing conflicts between different age groups, communication breakdowns) [27,45,58].

On the one hand, age diversity within virtual teams can contribute to promoting innovative ideas and ultimately allow virtual, diverse teams to outperform regular ones [58]. Moreover, virtual workplaces enable employees to contribute to the organization’s strategic goals regardless their time zone, which can nourish their motivation and help the organization to maintain high productivity [55].

On the other side, scholars have raised some concerns towards the quality of coworkers relationships and informal interactions [27,58], work-life conflict and general satisfaction of belongingness motives [27], organizational commitment [45], and communication barriers [58]. Moreover, there might be age differences in the way these challenges are evaluated [55], and challenges related to a growing discrimination of older workers [34,35,58,59,60,61].

Raišienė and colleagues [55] found that, among 436 Lithuanian remote workers, older workers feel more comfortable collaborating in a physical space, while younger employees see themselves as more technologically-savvy and generally evaluated the positive implications of remote work (e.g., opportunity to learn new skills) [55]. Similar results were found by Portillo and colleagues [57] on a population of 4589 Basque teachers: older teachers felt less technologically competent than younger ones. Moreover, younger teachers showed more homogeneous scores in their level of digital competences compared to older ones (i.e., a standard deviation of 3.03 points for juniors vs. 4.25 points for seniors), suggesting wider differences in digital competences among seniors [57].

Moreover, the more prominent role of technology to perform work activities has tendentially led to increased discrimination of older workers, perceived as less technologically savvy [58,59]. Generalizations concerning older workers’ resistance to technology have been confirmed as a very common stereotype even before the pandemic [62]. Furthermore, in crisis situations, age stereotypes can worsen as a result of policies and public conversations portraying the pandemic as an “older adult’s problem”, which essentially nourishes an imaginary barrier between juniors and seniors [34,35,60,61]. However, despite the stereotypes, data showed a different situation. In fact, evidence from the U.S. indicated that about 75% of the older internet users aged 65+ go online every day, displaying eagerness to learn and apply new skills where needed [33]. Nonetheless, in the context of the COVID-19 pandemic, the aforementioned aspects (i.e., pervasiveness of technology in work activity leading to negative perceptions of older workers’ abilities, policies, and narratives portraying the pandemic as an “older adult’s problem”) can foster specific age stereotypes that, in turn, can increase communication barriers and conflicts among colleagues of different age groups and decrease the willingness to work together [58]. A failure in communication and collaboration will eventually decrease knowledge transfer processes within the organization, though doing so is fundamental for organizations to endure high levels of ambiguity in times of crisis and effectively handle change [58,63]. As a result, organizational outcomes and performance can be negatively affected. As highlighted by Raišienė and colleagues [55], leaders can play a major role in their ability to promote positive interactions and foster mutual collaboration between employees despite their age. Clearly, more research is needed to deepen our understanding of age stereotypes contextualized in crisis situations and clarify the role that technology is progressively occupying in our society.

Furthermore, much of the literature addressing virtual collaboration during this pandemic refers to workers categorized into generations, while conversations revolving around the concept of “generation” should be used and examined carefully [64]. In fact, according to Rudolph and Zacher [59,64], in the COVID-19 context generational conversations can further stress a division between juniors and seniors. An example is the hashtag #BoomerRemover that became popular on social media as a nickname for the pandemic and has been pointed out by several authors [33,34,35,49,60]. Therefore, the adoption of a lifespan development framework is preferable to appreciate the effect of COVID-19 on different age groups [64].

To summarize, virtual workplaces have come with specific opportunities (i.e., age diversity can stimulate innovation, possibility to work despite the time zone, increased productivity) and challenges (e.g., work-life conflict, satisfaction of belonginess motives, the exacerbation of existing conflicts between different age groups, communication breakdowns). In relation to challenges, authors highlighted several aspects building on age-related differences in perceptions and discrimination processes towards older workers. Ignoring these challenges can hinder the progress of opportunities (e.g., resulting in communication breakdowns and lower organizational performance).

### 3.4. Key Individual and Organizational Resources and Strategies, and Age

According to Hu [54], the COVID-19 pandemic reflects a crisis-opportunity dialectic. In fact, the author explained that in Chinese, “crisis” encapsulates both the meanings “danger” and “opportunity”. On the one hand, individuals can engage in different strategies leveraging personal resources such as emotional regulation, work ability, and self-efficacy. On the other hand, organizations can step forward by implementing targeted interventions to support workers, helping them acquire personal resources (e.g., self-efficacy) and/or introducing supportive contextual factors (i.e., job autonomy). Age has been shown as an important aspect to consider in analyzing and designing strategies and interventions (e.g., job crafting and gratitude interventions, High Involvement Management practices).

Despite the lack of evidence, relevant theoretical contributions have been developed providing suggestions on how to deal with the negative aspects of the pandemic and increase the odds to seize the opportunities.

#### 3.4.1. Key Individual Resources and Strategies

The global pandemic took a toll on individuals’ wellbeing, severely affecting the labor market and economic context, and increasing the levels of uncertainty [40]. Scholars used pre-pandemic evidence to explain the resources (e.g., control over one’s emotions) and strategies (e.g., self-regulation) used—or that may be used—by workers to deal with COVID-19-related challenges.

Grote and Pfombreck [40] postulated the role of future time perspective in uncertainty management as a key component to ensure successful aging amidst this crisis. Building on Griffin and Grote’s [65] uncertainty regulation framework, they argued the longer the future pictured by individuals, the greater the implied levels of uncertainty. In fact, an extended time frame means more unpredictability. With advancing chronological age, the perceptions of remaining time to capitalize on opportunities are reduced [40]. However, personal resources such as emotion regulation can widen these perceptions. In this regard, Grote and Pfombreck [40] presented evidence of a greater emotion regulation capacity of older workers. This could explain their higher tolerance to unpredictability and greater engagement in self-regulation strategies. Through these self-regulatory processes, workers can align their endogenous (i.e., internal) uncertainty to the exogenous (i.e., external) one engaging in either opening (i.e., career change decision making such as switching job or anticipating retirement) or closing (i.e., looking for stability and predictability, such as continuing performing their regular job) behaviors [40]. Therefore, by regulating the amount of experienced uncertainty, individuals can promote a more successful aging fostering an open-ended future time perspective [40], despite the disruptive effect that the pandemic had on future opportunities.

The greater emotion regulation capacity of older workers has also been supported by Kooij [41] to explain differences between juniors and seniors’ responses to COVID-19′s consequences. According to the author, the current pandemic is a normative history-graded event, potentially triggering person-environment misfit, depleting individuals’ personal resources, and threatening the work sphere. However, older workers seem to be generally more comfortable in dealing with adversities. Therefore, they respond through engaging in different self-regulations strategies to maintain or restore their fit with the environment. These strategies can involve adaptive or proactive levels of goal engagement and disengagement, functional to the situation [41]. Proactive actions are self-initiated and can consist either of a goal pursuit (i.e., goal engagement, such as upgrading digital competences) or of a withdrawal (i.e., goal disengagement, such as reflecting on past experiences). Adaptive strategies are ascribable to coping strategies, such as asking for help (i.e., goal engagement) or cognitively restructuring the importance of a skill to avoid taking on a new training (i.e., goal disengagement). Due to their less cumulated experience and personal resources, younger workers seem to struggle more in effectively engaging in these strategies [41].

Nonetheless, older workers are a very heterogeneous population: more specifically, the extent to which older workers engage in self-regulation strategies and the type of adopted strategy also depend on several inter-individual differences (e.g., level of personal resources) [41]. According to Kooij [41], this variability might explain why after the pandemic, some older workers decided to anticipate retirement, some decided to postpone it, and others (especially in the healthcare sector) decided to come back to work while already in retirement.

Work ability (i.e., a person’s ability to continue working, considering the physical, mental, social, environmental, and organizational demands of their job) is another important factor influencing workers’ decision whether to remain in the workforce, in particular for older ones [44]. However, as underlined by Truxillo and colleagues [44], work ability encourages not only older workers’ decisions and behaviors during the pandemic, but also those of other people with vulnerabilities (e.g., underlying conditions). Therefore, it is a key factor influencing everyone’s wellbeing at work. In the context of the pandemic, work ability has largely been influenced by personal responses to COVID-19 itself (e.g., beliefs surrounding one’s own vulnerability) and by ageist narratives that might perpetrate a vulnerable image of older individuals. Furthermore, work ability generally decreases with the progression of chronological age. Therefore, work ability is likely to be damaged by a pandemic x health x age interaction [44]. The depletion of one’s work ability is likely to increase turnover and retirement, especially across those older and vulnerable populations [44]. For this reason, it is crucial to keep ensuring good work ability.

In essence, personal resources and strategies hold a major role in the context of the COVID-19 pandemic, influencing workers’ responses to its consequences. However, there is variability in personal resources and strategies held and used by individuals of different age. For example, older workers seem to engage more in self-regulation strategies promoted by a greater emotion regulation capacity. At the same time, older workers have a higher risk to experience poor work ability, as a result of personal beliefs surrounding one’s own vulnerability (i.e., higher perceived risk) and of ageist narratives (i.e., older workers are frailer). In turn, work ability affects the return-to-work phase and influences decisions about retirement.

#### 3.4.2. Key Organizational Resources and Strategies

Organizations can help workers of different ages in dealing with the pandemic and its consequences. On one side, organizations can provide specific interventions targeting personal resources (i.e., positive emotions, proactive and adaptive responses, work ability, resistance to change, and self-efficacy) [40,41,44,58]. On the other side, organizations can design and introduce contextual/work-related factors supporting an age-diverse workforce in dealing with the pandemic (i.e., job autonomy) [41]..

For instance, based on Luthans and colleagues [66] PsyCap (i.e., psychological capital) theory and PCI (i.e., psychological capital intervention), Kooij [41] suggested to explore the development of positive emotions implementing micro-interventions promoting gratitude or personal resources such as hope, optimism, efficacy, and resiliency [41]. The author, in fact, reported findings from Losada-Baltar and colleagues [67] showing that personal resources providing positive emotions can help younger workers or older workers with low resources to deal better with the COVID-19 consequences (e.g., career shocks, lockdown, and isolation).

To encourage the adoption of proactive or adaptive responses to unforeseen situations, organizations can implement job crafting interventions [41]. For example, there is evidence showing the effectiveness of a crafting towards strengths’ training in promoting self-regulation strategies among older workers rather than younger ones, through the enhancement of their person-job fit (i.e., the degree of compatibility between the employees and their job and job tasks) [41,68].

In addition, Truxillo and colleagues [44] suggested several strategies to maintain or restore work ability, which influences people’s decision whether to return or remain at work and whose levels might have been decreased by the current pandemic. According to the authors, examples of actions that the organization can undertake are: providing employees with more control over their work schedule, fostering support between coworkers and supervisors, and implementing more stringent safety protocols. Improving work ability can help workers of all ages, but especially older workers to keep working productively despite the challenges (e.g., the sudden implementation of remote work).

Moreover, employees’ resistance to change such as skepticism about working from home could be overcome by implementing job redesign interventions promoting a transition to remote work [48]. In fact, a cross-sectional study on 739 Greek workers showed that one third of the participants (34.4%) experienced teleworking, but more than half (52.1%) were not willing to work remotely [69]. Hence, providing the adequate resources to deal with these new ways of work is essential [41,58]. This could be more easily achieved when managers endorse a transformational leadership style [58] and adopt High Involvement Management (HIM) practices [41]. On the one hand, transformational leaders are encouraged to communicate the remote work’s implementation, anticipating expectations and promoting a stereotype-free working environment [58]. On the other hand, HIM practices have been shown to foster more collaborative relationships within virtual settings [41]. Together, they would benefit especially older workers’ wellbeing, allowing them to continue working in a stereotype-free and supportive working environment.

In particular, HIM practices targeting employees’ self-efficacy are thought to motivate workers of all ages to remain active in the job market [41]. Examples of actions that organizations can undertake are: (i) providing employees with developmental opportunities (e.g., trainings increasing digital competences for older adults), (ii) acknowledging older workers’ role in helping younger coworkers dealing with the uncertainties originated by the COVID-19 pandemic (i.e., thus promoting information sharing among employees of different age and background [58]), and/or (iii) giving employees more autonomy over their work [41].

In particular, job autonomy has been shown to be a key contextual/work-related factor significantly expanding future time perspective. As highlighted by Grote and Pfombreck [40] in their commentary, open-ended future time perspectives can promote older workers’ motivation to continue working, promoting their successful aging at work, potentially pushing them to postpone retirement. Future time perspective has been linked to higher job search self-efficacy, which can be particularly useful in time of crisis for both younger and older workers [40,70]. Therefore, organizations are advised to give employees more discretion over their work [41].

To conclude, organizations can adopt different interventions targeting different personal and contextual aspects of individuals and their relationship with work during the pandemic (e.g., positive emotions, job autonomy). Moreover, workers of different age might benefit from different types of strategies. For instance, older workers make the most of strategies such as crafting towards strengths interventions, work ability interventions, and trainings for digital competences, while younger workers benefit more from personal resources interventions or when they are flanked by older colleagues.

## 4. Discussion

The scope of this review was to analyze and summarize the different scientific contributions about workplace aspects of the COVID-19 pandemic, while considering potential age-differential effects.

Our findings highlighted an age-related effect of the current pandemic, to which individuals responded differently depending on their age.

First, the intertwined effects between COVID-19, occupation-specific risks, and the role of age have been addressed. Occupation-specific risk in the context of the pandemic has been defined across two elements (i.e., frequency of exposure to potential diseases and physical proximity to others) [20]. Findings supported the idea that occupation-specific risks increase their severity when older workers are those exposed [24,27]. Examples of occupations with high risks are low-skill low-income occupations (e.g., construction) or essential occupations (e.g., healthcare) [20,21,22]. 

Subsequently, regarding the impact that COVID-19 had on the labor market, we found that younger workers showed the higher share of job loss [30,31,32] and the higher levels of job insecurity [37,39] and turnover intentions [22,37,42,43]. However, more complex pathways branched off as soon as we considered further data. In fact, younger workers’ job losses’ rate stabilized quicker than older workers [21,29,33], which in turn faced greater barriers for reemployment (i.e., age stereotypes) [27,29,31,34,35]. Fewer employment opportunities are among the relevant contextual factors (i.e., others are perceptions of discrimination and financial situation) [28,31,32,45] influencing older workers’ retirement intentions. In addition to contextual factors, researchers showed that individual ones (i.e., work ability, job importance, perception of threat) can influence workers’ decisions whether to continue working [27,28,34,44]. Different combinations of factors can push older workers to anticipate or postpone retirement. A third retirement-related phenomenon that has been observed as a result of the COVID-19 pandemic consists of retired healthcare workers reentering the workforce, to help healthcare institutions dealing with the workload [26,48,49].

Next, we reviewed the impact that COVID-19 had on remote work in light of specific opportunities (i.e., promoting organizations’ agility and flexibility, global talents’ attraction, lower real estate costs, protection of vulnerable workers, sustainability) [29,45,55] and challenges (i.e., heterogeneity across occupations, exacerbating existing inequalities, worries around the activity) [29,34,56]. We found contradictory results suggesting that both younger and older workers’ opportunities to work from home can be reduced depending on the type of job they are employed in (e.g., low-skill manual jobs) [34,56]. Moreover, older workers hold generally more negative perceptions on the experience of remote work [55]. Within virtual spaces of collaboration, the results supported the existence of communication breakdowns and tensions between younger and older workers [27,58]. This conflict may also be affected by negative age stereotypes against older workers (i.e., low technological savviness, resistance, frailty, and focus of the pandemic’s inconveniences) perpetrated by organizations and governments [34,35,58,59,60,61,64]. Despite these stereotypes, research results suggested that older workers are as able as younger ones to work from home.

Lastly, the commentaries of several authors contributed to the understanding of age differences in response to historical events. In fact, older workers are thought to respond more effectively to the negative effects of the pandemic engaging in different self-regulation strategies of proactive or active goal (dis)engagement [40,41]. This might be due to their greater emotion regulation capacity [40]. The engagement in those strategies help workers to deal with the pandemic-related unpredictability, fostering an open-ended future time perspective and promoting a more successful aging at work [40,41]. However, older workers also have lower perceptions of work ability, which is an important determinant in the return-to-work phase and influences decisions about retirement [44]. Organizations can help workers developing personal resources in several ways (e.g., implementing job crafting intervention to promote self-regulation strategies and positive emotions, HIM practices enhancing self-efficacy, fostering support between coworkers and supervisors to maintain work ability) [40,41,44]. Moreover, organizations can introduce supportive contextual/work-related factors such as providing workers with more job autonomy: in fact, job autonomy is related to greater future time perspectives and therefore higher motivation to continue working [40,41].

It is important to acknowledge that there are some limitations. First, more than half of the articles were commentaries or perspectives, and this limits the empirical foundation for evidence-based conclusions. Second, the reviewed articles were very heterogeneous from a methodological and clinical (i.e., participants) point of view. This led to sometimes contradictory results even within the same country and timeframe (i.e., Monahan and colleagues [34] and Moen and colleagues [31] data on U.S. filed early retirement applications as a consequence of the pandemic). Overall, we obtained a good country representativeness: in fact, studies were from 18 different countries, including low-income countries. However, this country representativeness was not equally distributed across the analyzed topics, therefore some findings (e.g., data on retirement) are very country-specific (i.e., United States) and not generalizable. Lastly, we found a limited number of studies delving topics such as job insecurity, with a lack of a common definition over this concept. Therefore, evidence is not fully conclusive.

On the basis of these findings, several considerations for future research and practical implications are necessary.

In the short term, research should further investigate the pandemic x occupation x age interactions to support a safe workforce reentry and ensure good working conditions for all workers. Therefore, we advise future studies to consider the concrete effect of the COVID-19 pandemic on workers’ perceptions and behaviors. For example, we need more understanding on the differential impact according to workers’ sector and occupations. In this respect, further determinants such as education or socioeconomic status should be considered. Furthermore, great attention should be given to reemployment barriers determinants such as age stereotypes, and changes in their prevalence due to the pandemic. The role of personal determinants in reemployment decisions and differences across age should be investigated as well. For instance, perceptions of job search self-efficacy (i.e., personal beliefs of being able to successfully look for a new job and obtain employment [71]) might promote positive or negative job search expectations [27], ultimately influencing the willingness to engage in the job search and potentially hindering reemployment. Lastly, other concepts beyond chronological age, such as subjective age or perceptions of aging, might be accounted for as control variables.

In the long term, more research is needed on the personal and contextual determinants that support workers of different age groups in dealing with major critical life events. In this respect, future studies might include the age-differential impact of organizational policies and strategies to manage the pandemic [59]. To provide robust data, longitudinal studies or interrupted time series design are suggested. Lastly, the long-term consequences of prolonged work from home and physical distance on work-related processes and outcomes are still to be fully explored [59]. In this respect, cross-cultural studies might be a valid alternative to increase our understanding of the determinants promoting different trajectories and results. Moreover, a deeper understanding is needed on the barriers limiting older workers from reentering the workforce and for designing effective reemployment interventions.

From a practical standpoint, the findings of this review suggest that organizations and policy makers should focus on individual (e.g., work ability, positive emotions) and contextual (e.g., occupational risk, job autonomy) factors to support workers of different ages in dealing with the current pandemic, and possible future disruptive events.

A risk-assessment tool that takes into account both age and occupation-specific risks, could be useful to plan a safe workforce integration of people with elevated risk of COVID-19 (See Larochelle [24]). Moreover, job redesign interventions could also support organizations in assigning vulnerable employees to job tasks that have smaller risk of exposure. This can be done through adapting remote work strategies that can also buffer the labor market disruption caused by COVID-19, reducing employees’ chances to be laid off.

In light of our findings, we encourage the development of tools such as reemployment interventions and workforce emergency savings plans, which provide greater financial security to workers that have been left out of the workforce.

Lastly, attention is needed towards the factors promoting a successful integration of younger and older workers within virtual workspaces. Our findings underlined the role of individual (i.e., older workers’ perceptions of age discrimination, younger workers’ negative view of older colleagues) and contextual (i.e., transformational leadership) factors that (i) influence virtual collaboration across age groups and (ii) guide older employees’ retirement intentions.

In conclusion, the articles revealed great variability across age groups in the impact this pandemic has had on employment and several work-related aspects (i.e., occupational risk, remote work). Findings supported the adoption of an age perspective in the interpretation of normative history-graded events such as the current pandemic, and highlighted different responses depending on workers’ age. On the one hand, older age influences (i) the severity of occupation-specific risks, (ii) unemployment by enhancing barriers for reemployment, (iii) retirement anticipation or postponing behaviors, (iv) negative perceptions on the experience of remote work, and (v) the ability to respond more effectively to the negative effects of the pandemic by engaging in different self-regulation strategies. On the other hand, younger age groups faced (i) greater job loss share but with a quicker rebound and (ii) higher job insecurity and turnover intentions, and at the same time (iii), younger employees hold negative age stereotypes against older workers triggering tensions and communication breakdowns.

## Figures and Tables

**Table 1:** List of papers selected for the scoping review. Papers are organized following a subsection-reference type-reference number order. Papers repeated in two or more subsections are highlighted in grey.

Subsection	Author(s)	Reference Type	Data Type and Source	Country	Key Result(s)
	[20] St-Denis (2020)	Empirical	Quantitative— (a) O*Net; (b) Canadian Census of the Population	Canada	Occupational risk (i.e., risk of exposure to COVID-19) is measured through physical proximity and frequency of exposure scores. Higher occupational risk for low-income low-skill occupations. Younger workers are more likely to have jobs with higher occupational risk.
3.1. Occupational Risk and Age	[21] Hoehn-Velasco, Silverio-Murillo, and de la Miyar (2021)	Empirical	Quantitative— Mexican Social Security Institute	Mexico	Higher COVID-19-related job loss for younger and low-income workers. A total of 1.1 million formal jobs lost in the first five months of the pandemic. Quicker recovery of younger workers from unemployment (i.e., regained stability) opposed to older workers’ reemployment difficulties.
	[22] Kim, Lee, and Cho (2020)	Empirical	Quantitative Data Collection (*n* = 377)	South Korea	Occupation-specific COVID-19-related risk of healthcare workers. Workers’ age was negatively related with turnover intentions.
	[25] Glynn (2020)	Empirical	Quantitative— Office for National Statistics	U.K.	Estimated projections on U.K.’s mortality rate among different age groups highlighted higher vulnerability of older individuals.
	[23] Ashcroft (2020)	Commentary/ Perspective	-	U.K., Italy	Higher mortality rate for workers aged 50 or older. In Italy, 74 doctors in their 60s died of COVID-19 by April 2020.
	[24] Larochelle (2020)	Commentary/ Perspective	-	China, United States	Higher mortality rate for older adults (e.g., 3.6% for those aged 60–69). Framework to evaluate individual’s COVID-19-related risk to continue working based on occupational risk and likeability of death.
3.1. Occupational Risk and Age	[26] Sabath and Colt (2020)	Commentary/ Perspective	-	United States	Mortality rate escalated when 60-year-olds were compared to the 70-year-olds. Higher mortality is related with age and sector (i.e., 37% of U.S. deaths in healthcare workers were aged 65 years or more). Remote work or roles avoiding facing patients are advised for those retired workers reentering the workforce.
	[27] Kanfer, Lyndgaard, and Tatel (2020)	Commentary/ Perspective	-	Global Scale	Higher mortality rate and occupational risk for workers aged 50 or older. The pandemic increased reemployment barriers for older workers. Due to the pandemic, by April 2020, 49% of the U.S. workforce was working remotely. Concerns such as satisfaction of belonginess motives are raised.
	[28] Settersten Jr et al. (2020)	Commentary/ Perspective	-	Global Scale	Occupational risks increase with age. Older workers’ retirement intentions are influenced by personal (i.e., perception of threat) and contextual (i.e., financial situation) factors.
	[21] Hoehn-Velasco, Silverio-Murillo, and de la Miyar (2021)	Empirical	Quantitative— Mexican Social Security Institute	Mexico	Higher COVID-19-related job loss for younger and low-income workers. A total of 1.1 million formal jobs lost in the first five months of the pandemic. Quicker recovery of younger workers from unemployment (i.e., regained stability) opposed to older workers’ reemployment difficulties.
3.2. Labor Market Implications and Age	[22] Kim, Lee, and Cho (2020)	Empirical	Quantitative Data Collection (*n* = 377)	South Korea	Occupation-specific COVID-19-related risk of healthcare workers. Workers’ age was negatively related with turnover intentions.
	[29] Almeida and Santos (2020)	Empirical	Quantitative— Portuguese National Institute of Statistics	Portugal	Quicker recovery of younger workers from unemployment (i.e., regained stability) opposed to older workers’ reemployment difficulties. Unemployment influences job insecurity, which affects turnover intentions. Some occupations (e.g., low-income) struggled transitioning online.
	[30] Kikuchi, Kitao, and Mikoshiba (2021)	Empirical	Quantitative – (a) Labor Force Survey; (b) Monthly Labor Survey; (c) Empl. Status Survey	Japan	Higher pandemic-related unemployment for workers for low-income jobs. Higher COVID-19-related job losses for younger workers.
	[31] Moen, Pedtke, and Flood (2020)	Empirical	Quantitative— Current Population Survey (IPUMS)	United States	Higher COVID-19-related job loss for younger. Greater reemployment barriers for older workers (e.g., age discrimination). Data on filed early (i.e., April 2020) retirement requests showed little increase in retirement as a consequence of the COVID-19 pandemic.
3.2. Labor Market Implications and Age	[32] Sharmeen and Ahmed (2020)	Empirical	Quantitative Data Collection (*n* = n.d.)	Bangladesh	About 5% of the participants in the study lost their current job, almost 10% lost one of their current jobs, and approximately 9% saw their job offers postponed as a result of the pandemic. Older workers’ retirement intentions come from fewer hiring opportunities.
	[37] Bajrami et al. (2020)	Empirical	Quantitative Data Collection (*n* = 624)	Serbia	Negative correlation between job insecurity and age. Positive correlation between job insecurity and turnover intentions. Older workers’ lower turnover intentions.
	[39] Ranta, Silinskas, and Wilska (2020)	Empirical	Quantitative Data Collection (*n* = 222)	Finland	Younger participants (18–29) showed to be more worried about the consequences of the pandemic on their career than older one (30–65).
	[42] Yáñez et al. (2020)	Empirical	Quantitative Data Collection (*n* = 303)	Peru	Older workers showed lower levels of turnover intentions.
	[43] Zhang et al. (2020)	Empirical	Quantitative Data Collection (*n* = 240)	Bolivia	Older workers showed lower levels of turnover intentions.
	[46] Biggs (2021)	Empirical	Quantitative— Social Security Administration’s Office of the C.Act.	United States	Analyses on Social Security retirement benefits pandemic-related consequences indicated a 9% reduction for individuals born in 1960 and after.
	[26] Sabath and Colt (2020)	Commentary/ Perspective	-	United States	Mortality rate escalated when the 60-year-olds were compared to 70-year-olds Higher mortality is related with age and sector (i.e., 37% of U.S. deaths in healthcare workers were aged 65 years or more). Remote work or roles avoiding facing patients are advised for those retired workers reentering the workforce.
	[27] Kanfer, Lyndgaard, and Tatel (2020)	Commentary/ Perspective	-	Global Scale	Higher mortality rate and occupational risk for workers aged 50 or older. The pandemic increased reemployment barriers for older workers. Due to the pandemic, by April 2020, 49% of the U.S. workforce was working remotely. Concerns such as satisfaction of belonginess motives are raised.
	[28] Settersten Jr et al. (2020)	Commentary/ Perspective	-	Global Scale	Occupational risks increase with age. Older workers’ retirement intentions are influenced by personal (i.e., perception of threat) and contextual (i.e., financial situation) factors.
3.2. Labor Market Implications and Age	[33] Morrow-Howell, Galucia, and Swinford (2020)	Commentary/ Perspective	-	Global Scale	Quicker recovery of younger workers from unemployment (i.e., regained stability) opposed to older workers’ reemployment difficulties. Older workers’ retirement intentions are affected by the pandemic effect on their finances. A total of 75% of U.S. internet users aged 65+ go online every day.
	[34] Monahan et al. (2020)	Commentary/ Perspective	-	Global Scale	Older workers (aged 65+) saw a 2.9% increase in their unemployment rate from April 2019 to April 2020 and faced greater barriers for reemployment. Older workers’ retirement intentions are influenced by personal (i.e., perception of threat) factors. Early retirement increased by 7% in April 2020. Older workers have less opportunities for remote work because they are more likely to be employed in manual jobs.
	[35] Berridge and Hooyman (2020)	Commentary/ Perspective	-	Global Scale	Ageism negatively affects older workers’ reemployment. Due to the pandemic, negative stereotypes towards older workers increased and negatively affected collaboration in virtual workspaces.
	[40] Grote and Pfrombeck (2020)	Commentary/ Perspective	-	Global Scale	Self-regulation strategies to manage uncertainty influence future time perspectives and differ across age. Older workers are thought to adopt more effectively those strategies.
	[41] Kooij (2020)	Commentary/ Perspective	-	Global Scale	Emotional regulation strategies to keep P-E fit in challenging environments. Older workers are thought to adopt more effectively those strategies.
	[44] Truxillo, Cadiz, and Brady (2020)	Commentary/ Perspective	-	Global Scale	The impact of the pandemic on work ability is analyzed, along with its repercussions on older workers’ retirement intentions.
3.2. Labor Market Implications and Age	[45] van Dalen and Henkens (2020)	Commentary/ Perspective	-	Global Scale	Financial setbacks due to the pandemic influence retirement intentions. Remote work limits face interaction but improves organizations’ flexibility.
	[48] Peisah et al. (2020)	Commentary/ Perspective	-	Global Scale	Retired healthcare professionals coming back to work should avoid patient-facing tasks and prefer a remote contribution.
	[49] Previtali, Allen, and Varlamova (2020)	Commentary/ Perspective	-	Global Scale	Retired healthcare professionals returning to work face additional risks due to their age and occupation-specific characteristics. Pandemic-related ageism negatively affects collaboration in virtual settings.
	[29] Almeida and Santos (2020)	Empirical	Quantitative— Portuguese National Institute of Statistics	Portugal	Quicker recovery of younger workers from unemployment (i.e., regained stability) opposed to older workers’ reemployment difficulties. Unemployment influences job insecurity, which affects turnover intentions. Some occupations (e.g., low-income) struggled transitioning online.
3.3. Remote Work and Age	[55] Raišienė et al. (2020)	Empirical	Quantitative Data Collection (*n* = 436)	Lithuania	During the lockdown, workers applying telework swelled to 40%, compared to 13% in 2017. Remote work has advantages (e.g., lower real estate costs). Older workers worry more about the disadvantages (e.g., work life conflict). Transformational leadership can facilitate remote work and collaboration.
	[56] Gallacher and Hossain (2020)	Empirical	Quantitative—Canada’s Employment Income Statistics	Canada	A total of 41% of jobs in Canada have the potential to be done from home. Less possibility for remote work was linked to higher job loss rate. Younger workers had lower levels of remote work opportunities.
	[57] Portillo et al. (2020)	Empirical	Quantitative Data Collection (*n* = 4589)	Basque Country	Older teachers feel less technologically competent than younger ones. Younger teachers showed more homogeneous scores than older ones.
	[27] Kanfer, Lyndgaard, and Tatel (2020)	Commentary/ Perspective	-	Global Scale	Higher mortality rate and occupational risk for workers aged 50 or older. The pandemic increased reemployment barriers for older workers. Due to the pandemic, by April 2020, 49% of the U.S. workforce was working remotely. Concerns such as satisfaction of belonginess motives are raised.
	[34] Monahan et al. (2020)	Commentary/ Perspective		Global Scale	Older workers (aged 65+) saw a 2.9% increase in their unemployment rate from April 2019 to April 2020 and faced greater barriers for reemployment. Older workers’ retirement intentions are influenced by personal (i.e., perception of threat) factors. Early retirement increased by 7% in April 2020. Older workers have less opportunities for remote work because they are more likely to be employed in manual jobs.
	[35] Berridge and Hooyman (2020)	Commentary/ Perspective		Global Scale	Ageism negatively affects older workers’ reemployment. Due to the pandemic, negative stereotypes towards older workers increased and negatively affected collaboration in virtual workspaces.
3.3. Remote Work and Age	[45] van Dalen and Henkens (2020)	Commentary/ Perspective		Global Scale	Financial setbacks due to the pandemic influence retirement intentions. Remote work limits face interaction but improves organizations’ flexibility.
	[58] Urick (2020)	Commentary/ Perspective		Global Scale	Remote work brought opportunities (i.e., innovation) and challenges (i.e., exacerbation of age-groups conflicts). Due to the pandemic, negative stereotypes towards older workers increased and negatively affected collaboration in virtual workspaces.
	[59] Rudolph and Zacher (2020)	Commentary/ Perspective		Global Scale	Older workers are perceived less technologically savvy. The pandemic exacerbated generational conversations and division between juniors and seniors (e.g., #BoomerRemover).
	[60] Ayalon (2020)	Commentary/ Perspective		Global Scale	The pandemic exacerbated the division between juniors and seniors (e.g., #BoomerRemover). This negatively affected virtual collaboration.
	[61] Harper (2020)	Commentary/ Perspective		Global Scale	The pandemic increased negative stereotypes towards older workers (e.g., frailer and less technologically savvy).
	[64] Rudolph and Zacher (2020b)	Commentary/ Perspective		Global Scale	The pandemic increased negative stereotypes towards older workers (e.g., frailer and less technologically savvy). The pandemic exacerbated generational conversations and division between juniors and seniors (e.g., #BoomerRemover).
	[55] Raišienė et al. (2020)	Empirical	Quantitative Data Collection (*n* = 436)	Lithuania	During the lockdown, people teleworking swelled to 40%, compared to 13% in 2017. Despite remote work advantages (e.g., lower real estate costs), older workers worry more about the disadvantages (e.g., work life conflict). Transformational leadership can facilitate remote work and collaboration.
	[57] Portillo et al. (2020)	Empirical	Quantitative Data Collection (*n* = 4589)	Basque Country	Older teachers feel less technologically competent than younger ones. Younger teachers showed more homogeneous scores than older ones.
	[69] Vatavali et al. (2020)	Empirical	Quantitative Data Collection (*n* = 730)	Greece	Results showed that 8.9% of the participants lost their job because of COVID-19.
3.4. Key Individual and Organizational Resources and Strategies, and Age	[40] Grote and Pfrombeck (2020)	Commentary/ Perspective		Global Scale	Self-regulation strategies to manage uncertainty influence future time perspectives and differ across age. Older workers are thought to adopt more effectively those strategies
	[41] Kooij (2020)	Commentary/ Perspective		Global Scale	Emotional regulation strategies to keep P-E fit in challenging environments. Older workers are thought to adopt more effectively to those strategies.
	[44] Truxillo, Cadiz, and Brady (2020)	Commentary/ Perspective		Global Scale	The impact of the pandemic on work ability is analyzed, along with its repercussions on older workers’ retirement intentions.
	[48] Peisah et al. (2020)	Commentary/ Perspective		Global Scale	Retired healthcare professionals coming back to work should avoid patient-facing tasks and prefer a remote contribution.
	[58] Urick (2020)	Commentary/ Perspective		Global Scale	Remote work brought opportunities (i.e., innovation) and challenges (i.e., exacerbation of age-groups conflicts). Due to the pandemic, negative stereotypes towards older workers increased negatively affecting collaboration in virtual workspaces.
	[59] Rudolph and Zacher (2020)	Commentary/ Perspective		Global Scale	Older workers are perceived less technologically savvy. The pandemic exacerbated generational conversations and division between juniors and seniors (e.g., #BoomerRemover).

## Data Availability

Not applicable.
